# Optimal mean arterial pressure and its objective statistical associations with clinical outcomes and multimodal monitoring cerebral physiology: A systematic scoping review

**DOI:** 10.14814/phy2.70545

**Published:** 2025-09-21

**Authors:** Rakibul Hasan, Angela Buchel, Kevin Y. Stein, Tobias Bergmann, Amanjyot Singh Sainbhi, Nuray Vakitbilir, Isuru Herath, Noah Silvaggio, Mansoor Hayat, Jaewoong Moon, Frederick A. Zeiler

**Affiliations:** ^1^ Department of Biomedical Engineering, Price Faculty of Engineering University of Manitoba Winnipeg Manitoba Canada; ^2^ Undergraduate Medical Education, Rady Faculty of Health Sciences University of Manitoba Winnipeg Manitoba Canada; ^3^ Department of Human Anatomy and Cell Science, Rady Faculty of Health Sciences University of Manitoba Winnipeg Manitoba Canada; ^4^ Section of Neurosurgery, Department of Surgery, Rady Faculty of Health Sciences University of Manitoba Winnipeg Manitoba Canada; ^5^ Department of Clinical Neuroscience Karolinska Institutet Stockholm Sweden; ^6^ Pan Am Clinic Foundation Winnipeg Manitoba Canada

**Keywords:** ABPopt, clinical outcome, MAPopt, multimodal monitoring, optimal pressure

## Abstract

Optimal mean arterial pressure (MAPopt), also known as optimal arterial blood pressure (ABPopt), represents a patient‐specific blood pressure range at which cerebral autoregulation is most intact. To date, literature on this personalized physiological target remains heterogeneous, scattered, and difficult to follow. This scoping review, following PRISMA‐ScR guidelines, examined studies that explored objective statistical associations between MAPopt and clinical outcomes and multimodal monitoring (MMM) cerebral physiology. Fifteen articles met the inclusion criteria for studies investigating the relationship between MAPopt and outcome, including seven neonatal studies, five cardiac arrest/surgery studies, two general ICU (Intensive Care Unit) studies, and one neurological ICU study. Fourteen of the fifteen studies found that maintaining blood pressure above or within MAPopt was linked to improved neurological outcomes, while pressures below MAPopt correlated with worse outcomes. In neonates with hypoxic–ischemic encephalopathy, deviations below MAPopt were associated with more severe brain injury. Similarly, cardiac arrest patients spending more time below MAPopt‐5 mmHg had higher mortality. Only one study evaluated MAPopt in relation to MMM data, identifying a nonlinear relationship between brain oxygenation and MAP deviation. This review underscores the need for further standardized research on MAPopt, particularly its interaction with MMM, to support its application in personalized critical care.

## 1 INTRODUCTION

Acute neurological injuries are considered a leading cause of death and disability worldwide (Zhong et al., 2025). Over the past several decades, significant progress has been made in achieving guideline‐based cerebral physiologic targets for managing moderate‐to‐severe brain injuries to improve outcomes (Stein et al., 2025; Zeiler et al., 2022). However, despite these advancements, outcomes have remained largely unchanged. This is likely due to the generalized approach of the current Brain Trauma Foundation (BTF) guidelines, which do not account for individual patient differences in secondary neural injury patterns and responses to treatment (Donnelly et al., 2019; Maas et al., 2017; Steyerberg et al., 2019). Current guideline‐based strategies do not address individual phenotypes, injury heterogeneity, and the dynamic nature of cerebral physiology (Stocchetti et al., 2017). As such, neurocritical care must move away from its current “one treatment fits all” paradigm and embrace personalized strategies tailored to the patient's individual physiologic needs. One promising approach is the development of personalized physiologic targets, which aim to optimize therapy based on real‐time patient data. Unlike fixed thresholds, such as those used in current guideline‐based management (Carney et al., 2017; Hawryluk et al., 2019), personalized targets allow for individualized, dynamic adjustments in care based on each patient's unique physiological state.

Multimodal monitoring (MMM) of brain physiological function has recently opened the door to the possibility of personalized subject‐specific physiologic metrics and targets, where various aspects of secondary neural injury pathways can be continuously monitored (Tas et al., 2022). MMM, which includes variables such as metabolism, perfusion, oxygenation, and electrical activity, has further emphasized the need for personalized and comprehensive neurocritical care (Casault et al., 2022). By integrating data from intracranial pressure (ICP), cerebral perfusion pressure (CPP), brain tissue oxygenation (PbtO_2_), and autoregulation indices, clinicians gain a more complete picture of a patient's cerebral physiology (Casault et al., 2022). This integrated approach is especially critical for managing traumatic brain injury (TBI), subarachnoid hemorrhage, and other acute neurologic conditions, where secondary injury can significantly affect outcomes. Secondary brain injury results in ongoing neuronal death in the days, months, or even years following the brain injury, and can significantly impede recovery (Maas et al., 2017). Since very little can be done to reverse primary brain injury, neurocritical care is almost exclusively limited to minimizing secondary injury. However, aside from raw physiological measures provided from the MMM devices, the signal sources can be further processed to derive live‐time measures of pressure‐flow physiology, as is the case with continuous cerebrovascular reactivity (CVR) monitoring (Zeiler et al., 2022; Zeiler, Ercole, Czosnyka, et al., 2020).

CVR refers to a critical mechanism through which cerebral blood vessels self‐regulate their tone in response to changes in cerebral perfusion pressure (CPP) to maintain a stable cerebral blood flow (CBF) despite changes in systemic pressure (Czosnyka et al., 1997). This process is crucial for protecting the brain from both hypo‐ and hyper‐perfusion‐related injury (Stein et al., 2025). CVR often becomes impaired in neurological conditions such as TBI, significantly reducing the range of systemic blood pressures over which this mechanism can keep CBF constant (Lassen, 1959; Toth et al., 2016). Thus, ensuring systemic and cerebral perfusion pressures are maintained in a range that facilitates constant and adequate CBF is key. Continuous CVR monitoring, through such measures as the pressure reactivity index (PRx) (Czosnyka et al., 1997), has facilitated the live‐time assessment of CVR at the bedside, receiving preclinical validation as measures of the autoregulatory curve (Sainbhi et al., 2021), and demonstrating strong associations with long‐term clinical outcomes in both TBI (Åkerlund et al., 2020; Zeiler et al., 2020) and subarachnoid hemorrhage (Svedung Wettervik et al., 2022) populations.

CVR monitoring can be further leveraged to derive continuously updating personalized blood pressure targets, such as optimal cerebral perfusion pressure (CPPopt) (Aries et al., 2012), or optimal mean arterial pressure (MAPopt) (Stein et al., 2025). MAPopt, also referred to as optimal arterial blood pressure (ABPopt) in the literature, refers to the optimal mean arterial pressure (MAP) at which CVR is best preserved. It is commonly calculated using noninvasive CVR metrics, such as COx (cerebral oximetry index) derived through near‐infrared spectroscopy (NIRS) (Gomez et al., 2022; Mathieu et al., 2020). When COx is plotted against MAP, the resulting curve typically forms a U‐shape. The lowest point (nadir) of this curve represents MAPopt—the pressure at which cerebral autoregulation is most intact. This MAPopt value, in theory, can be targeted by treating clinicians by manipulating systemic blood pressures to reach this MAP value intermittently or continuously. This MAPopt concept, particularly continuously derived versions, is of great interest not only in neurocritically ill populations, but also in general ICU (Gomez et al., 2025; Xie et al., 2025) and operating room (Beqiri et al., 2024; Stein et al., 2025) environments, where protection against secondary neural injury related to systemic hypoperfusion may lead to better long‐term cognitive outcomes in both ICU survivors and postoperative care environments.

Although MAPopt holds strong theoretical potential, it remains primarily a research tool. It has not yet been widely validated outside of investigational settings and is not currently a part of routine clinical practice, with significant knowledge gaps that remain. Literature to date on MAPopt/ABPopt remains heterogeneous, scattered within subspecialty publications, and at times hard to follow. This is particularly the case for continuously derived MAPopt/ABPopt measures, derived over entire care periods (i.e., ICU or operative), as continuous MAPopt/ABPopt measures are of primary interest for the future transition to personalized care. Specifically, the relationship between MAPopt and clinical outcomes is not fully understood, nor is the connection between MAPopt and multimodal cerebral physiological monitoring variables. Understanding how MAPopt correlates with pressure‐flow dynamics, oxygen and nutrient delivery, and other physiological processes is essential for clarifying its potential as a personalized therapeutic target. This scoping review aims to evaluate the existing literature on the objective statistical relationship between MAPopt and clinical outcomes, as well as its association with multimodal monitoring variables. In doing so, we will identify current knowledge gaps and highlight areas for future research to guide the next steps in the clinical application of MAPopt.

## 2 METHODS

This systematic scoping literature review was conducted following the Cochrane Handbook for Systematic Reviews methodology (Page et al., 2021). The information reported in this review conforms to the Preferred Reporting Items for Systematic Reviews and Meta‐Analyses extension for Scoping Reviews (PRISMA‐ScR) (Page et al., 2021; Tricco et al., 2018). The completed PRISMA‐ScR checklist can be found in the supplementary file S1. The objectives for this systematic scoping review search strategy were developed collaboratively by RH and FAZ.

### 2.1 Ethical consideration

All articles examined and included in this systematic scoping review were from previously published journals and are assumed to have been screened by these journals. Therefore, specific ethics approval for this systematic scoping review was not required.

### 2.2 Search question and criteria for inclusion and exclusion

Two questions examined in this review are as follows: (1) What are the associations between MAPopt and clinical outcome? (2) What are the associations between MAPopt and MMM cerebral physiology? To be included in this review, articles needed to include an objective, quantitative statistical comparison between MAPopt and clinical outcomes/MMM variables. For this study, MMM variables were defined as any raw or derived cerebral physiologic metrics/variables (i.e., ICP, CPP, PbtO_2_, cerebral NIRS, transcranial Doppler (TCD), CBF, CVR, compliance/compensatory reserve, autonomics, or entropy measures/variables). Studies that documented comparisons to other CPPopt or MAPopt/ABPopt variables derived from different CVR indices were not included in this review, as the focus was on association with MMM measures of other aspects of cerebral pressure–flow, nutrient, and oxygen delivery physiology. There was no restriction based on sample size or patient population for article inclusion. No animal studies were included.

The exclusion criteria of articles included articles that did not study MAPopt/ABPopt (this includes those that only make passive reference to MAPopt/ABPopt and focus only on upper and lower limits of regulation), articles that did not involve continuous multimodal monitoring techniques in comparison to MAPopt/ABPopt, and/or did not statistically compare MAPopt/ABPopt to either clinical outcome or MMM measures (as listed above), not peer‐reviewed or non‐full‐length articles (e.g., abstracts, theses, and conference papers), and non‐English articles.

### 2.3 Search strategy

Searches were conducted using BIOSIS, SCOPUS, EMBASE, MEDLINE, Global Health, and Cochrane Library, encompassing the period from the inception of each database until February 21st, 2025. Search strings were constructed, including terms and synonyms for MAPopt/ABPopt, multimodal monitoring, and clinical outcomes. Included in Appendix is the detailed search string used. Once a search was conducted using each database, the results were combined, and the papers were deduplicated to make an extensive list of potential articles. We conducted two different searches here: one search involved finding articles that include a relation between MAPopt and clinical outcomes, and the second search involved finding articles that include a relation between MAPopt and multimodal monitoring variables.

### 2.4 Selecting studies

After deduplication, the remaining articles were manually reviewed through a two‐stage, two‐reviewer approach. In the first stage, two reviewers (RH and AB) independently screened the titles and abstracts of each article based on the inclusion and exclusion criteria. Articles that were included went to the second stage of screening, where both reviewers looked at the complete article and assessed it based on the inclusion and exclusion criteria. Any disagreements between the reviewers were resolved by a third party (FAZ). The reference sections of the included articles were then screened to ensure that no relevant articles were missed during the search.

### 2.5 Data collection

Characteristics were recorded from each article included in this systematic scoping review, encompassing patient/subject information, general study information, and results. Patient/subject information included the patient cohort investigated, patient demographics, and sample size used in the study. General study information included the primary study objective, underlying pathology, technology used for CVR assessment, CVR index used for MAPopt derivation, and the relationship between MAPopt and outcome/MMM variables.

### 2.6 Statistical analysis

Considering the highly heterogeneous nature of the literature included, no formal meta‐analysis was performed.

### 2.7 Bias assessment

Given that the primary aim of this review was to conduct a comprehensive scoping review of the available literature, a formal bias assessment was not warranted.

## 3 RESULTS

### 3.1 Search strategy and results

For MAPopt versus outcome, a total of 155 articles were identified through the initial search, with 92 of these articles remaining after the deduplication process. These 92 articles were screened by title and abstract based on the inclusion and exclusion criteria. 41 of these articles were excluded for not meeting the inclusion criteria, leaving 51 articles that were screened by examining their full text. Of these 51 articles, 39 were excluded for not meeting the inclusion criteria, leaving 12 articles from the initial search included in this review. Through the examination of the reference sections of these included articles, an additional three articles were included, resulting in a total of 15 articles included in the “MAPopt versus clinical outcome” portion of the review.

**FIGURE 1:**
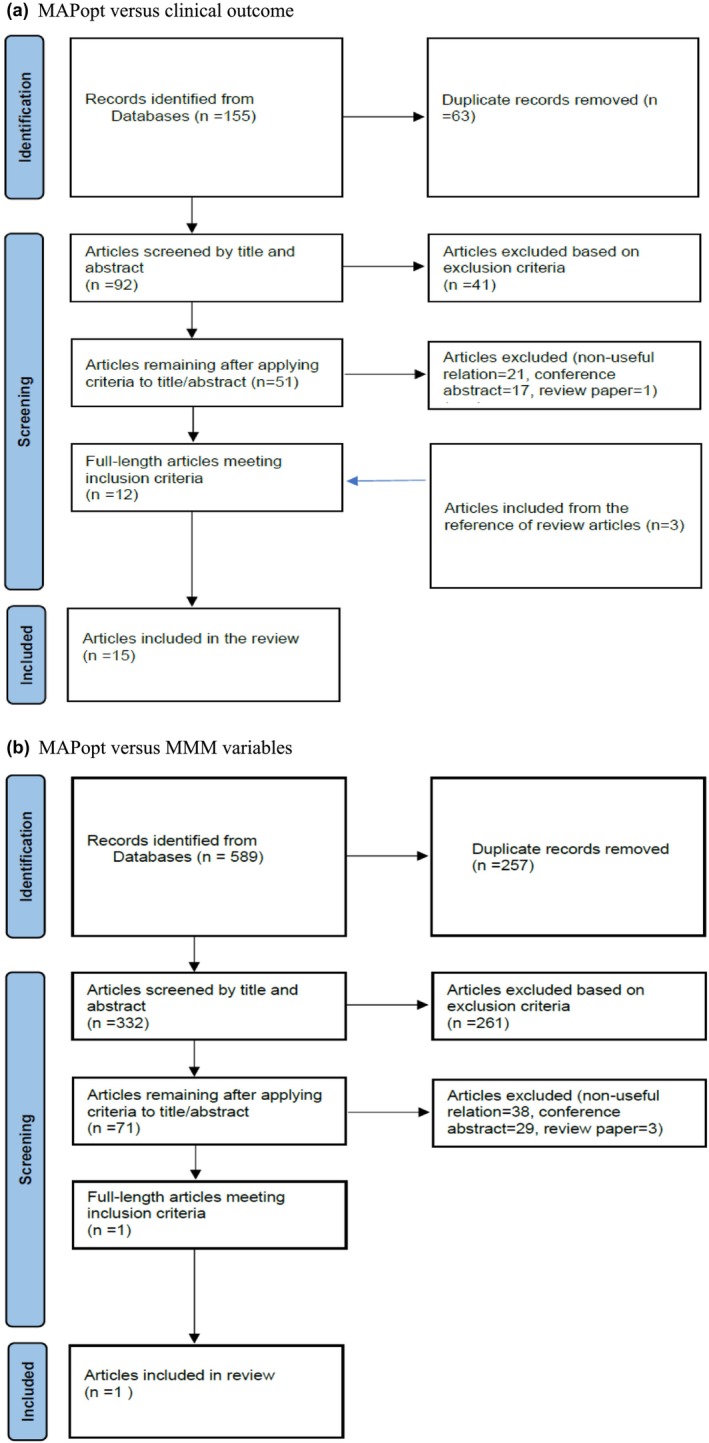
PRISMA flow diagrams of the systematically conducted scoping review. (a) MAPopt versus clinical outcome. (b) MAPopt versus MMM variables.

For MAPopt versus MMM variables, a total of 589 articles were identified through the initial search, with 332 of these articles remaining after the deduplication process. These 332 articles were screened by title and abstract based on the inclusion and exclusion criteria. 261 of these articles were excluded for not meeting the inclusion criteria, leaving 71 articles that were screened by examining their full text. Of these 71 articles, 70 were excluded for not meeting the inclusion criteria, leaving one article from the initial search that was included in this review. Through the examination of the reference sections of this article, no additional relevant article was found. This resulted in a total of one article being included in the “MAPopt versus MMM variables” part of the review. Figure 1 depicts the overall results for the searches and filtration processes using PRISMA flow diagrams.

### 3.2 Study demographics

For MAPopt versus clinical outcome, of the 15 articles included in the final review, seven involved neonates suffering hypoxic ischemic encephalopathy (Burton et al., 2015; Chavez‐Valdez et al., 2017; Howlett et al., 2013; Lee, Perin, et al., 2017; Lee, Poretti, et al., 2017; Liu et al., 2021; Tekes et al., 2015), five involved patients suffering from cardiac arrest or undergoing cardiac surgery (Griesdale et al., 2020; Hori et al., 2016; Kirschen et al., 2021, 2022; Lee et al., 2014), two studies involved general ICU patients (Khan et al., 2024; Zhang et al., 2024), and one study involved neurological ICU patients (Rivera‐Lara et al., 2019). NIRS was used to assess CVR in all the included studies except one (CVR was assessed invasively in this study). For MAPopt versus MMM variables, one article (Sekhon et al., 2019) was included in the final review. In this article, patients suffered from hypoxic‐ischaemic brain injury after cardiac arrest, and CVR was assessed invasively. Characteristics and relevant results from the individual studies are summarized in Tables 1, 2, 3, 4 (MAPopt vs. outcome) and Table 5 (MAPopt vs. MMM variables). Table 1 lists studies on the neonatal population, Table 2 lists studies on the cardiac arrest population, Table 3 lists studies on the general ICU population, and Table 4 lists studies on the neurological ICU population.

**TABLE 1:** Studies documenting statistical association between MAPopt and clinical outcome for the neonatal population.

Article	Patient cohort characteristics and study site	Primary objective of study	Underlying pathology	Technology used for CVR assessment	CVR index used for MAPopt derivation	MAPopt derivation process	MAPopt documented association with clinical outcome
Burton et al. (2015)	19 neonates Johns Hopkins Hospital Neonatal Intensive Care Unit	Investigating the relationship between acute autoregulatory vasoreactivity during treatment and neurodevelopmental outcomes at 2 years of age	Hypoxic–ischemic encephalopathy	Near‐infrared spectroscopy (NIRS) INVOS 5100 NIRS machine, Medtronic	Hemoglobin volume index (HVx) was used to derive MAPopt. Derivation of MAPopt was a single‐point measure.	Right and left HVx values were averaged and sorted into 5 mmHg bins of MAP to generate bar graphs. Bin with the most negative HVx was identified as MAPopt in each time period. When a nadir in HVx was not found, MAPopt was declared unidentifiable. Visual inspection was used to identify MAPopt values.	Children who developed impairments spent more time with blood pressure below MAPopt than children who did not develop impairments (*n* = 9; *p* = 0.048). Children with impairments had greater maximal blood pressure deviation below MAPopt (*p* = 0.019).
Chavez‐Valdez et al. (2017)	75 neonates (31 girls, 44 boys) Johns Hopkins Hospital Neonatal Intensive Care Unit	Investigating associations between cerebral autoregulation and cardiopulmonary injury in neonates with neonatal encephalopathy	Neonatal encephalopathy	NIRS INVOS 5100 NIRS machine, Medtronic	Hemoglobin volume index (HVx) was used to derive MAPopt. Derivation of MAPopt was a single‐point measure.	Right and left HVx values were averaged and sorted into 5 mmHg bins of MAP to generate bar graphs. Bin with the most negative HVx was identified as MAPopt in each time period. When a nadir in HVx was not found, MAPopt was declared unidentifiable. Visual inspection was used to identify MAPopt values.	Greater maximal blood pressure deviation above MAPopt during normothermia was associated with shorter duration of intubation in boys (*β* = − 0.142, *p* = 0.018); but during rewarming, it was associated with longer durations of intubated or noninvasive respiratory pressure support in girls (*β* = 0.033, *p* = 0.044). Greater maximal blood pressure deviation below MAPopt during normothermia was associated with a longer NICU stay in boys (*β* = 0.024, *p* = 0.018) but shorter stay in girls (*β* = −0.024, *p* = 0.024).
Howlett et al. (2013)	24 neonates Johns Hopkins University	Describe the relationship between autoregulation and neurologic injury in hypoxic–ischemic encephalopathy	Hypoxic–ischemic encephalopathy	NIRS Bilateral, adhesive, neonatal cerebral oximetry probes (INVOS), Medtronic	Hemoglobin volume index (HVx) was used to derive MAPopt. Derivation of MAPopt was a single‐point measure.	Right and left HVx values were averaged and sorted into 5 mmHg bins of MAP to generate bar graphs. Bin with the most negative HVx was identified as MAPopt in each time period. Visual inspection was used to identify MAPopt values.	MAP near or above MAPopt was associated with reduced neurologic injury, particularly in the paracentral gyri, white matter, basal ganglia, and thalamus. On the other hand, MAP deviation below MAPopt was associated with increased injury in those brain regions. Neonates with no, mild, or moderate/severe injury in paracentral gyri had median MAP deviations below MAPopt of 10 mmHg (5, 10 (IQR)), 15 mmHg (15, 20), and 15 mmHg (5, 15), respectively. For neonates with no, mild, or moderate/severe injury in basal ganglia, the median MAP deviations below MAPopt were 10 mmHg (5, 15), 12.5 mmHg (10, 15), and 15 mmHg (5, 15). Patients with no, mild, or moderate/severe injury in thalamus had median MAP deviations below MAPopt of 10 mmHg (5, 15), 10 mmHg (10, 15), and 15 mmHg (5, 15). Additionally, patients with minimal shifts in MAPopt between hypothermia and rewarming displayed better outcomes, suggesting that MAPopt stability may be predictive of lower injury severity.
Lee, Perin, et al. (2017)	75 neonates (44 boys, 31 girls) Johns Hopkins University	Investigated whether cerebral blood pressure autoregulation and kidney and liver injuries are associated in neonatal encephalopathy (NE).	Neonatal encephalopathy	NIRS INVOS 5100 NIRS machine, Medtronic	Hemoglobin volume index (HVx) was used to derive MAPopt. Derivation of MAPopt was a single‐point measure.	Right and left HVx values were averaged and sorted into 5 mmHg bins of MAP to generate bar graphs. Bin with the most negative HVx was identified as MAPopt in each time period. When a nadir in HVx was not found, MAPopt was declared unidentifiable. Visual inspection was used to identify MAPopt values.	Higher MAPopt during hypothermia was related to lower creatinine (*p* = 0.04). Among girls, higher MAPopt (*p* = 0.019) and longer blood pressure duration within MAPopt (*p* = 0.030) during hypothermia were associated with lower creatinine, suggesting a potential renal protective effect. During normothermia, time spent in MAP below MAPopt was associated with higher liver enzymes (*p* values are 0.04 and 0.02 for two hepatic enzymes), and during rewarming, time spent in MAP above MAPopt was associated with higher liver enzymes (*p* = 0.02), indicating hepatic stress.
Lee, Poretti, et al. (2017)	64 neonates (38 boys, 26 girls) Johns Hopkins University	Examined whether hemodynamic goals that support autoregulation are associated with decreased brain injury and whether these relationships are affected by birth asphyxia or vary by anatomic region	Hypoxic–ischemic encephalopathy	NIRS INVOS 5100 NIRS machine, Medtronic	Hemoglobin volume index (HVx) was used to derive MAPopt. Derivation of MAPopt was a single‐point measure.	Right and left HVx values were averaged and sorted into 5 mmHg bins of MAP to generate bar graphs. Bin with the most negative HVx was identified as MAPopt in each time period. When a nadir in HVx was not found, MAPopt was declared unidentifiable. Visual inspection was used to identify MAPopt values.	Greater duration and deviation of MAP below MAPopt were associated with greater injury in the white matter (deviation below: *p* = 0.047) and paracentral gyri (greater duration: 0.007; deviation below: 0.012). Spending more time within MAPopt resulted in less injury in white matter (*p* = 0.017) and basal ganglia (*p* = 0.04). Blood pressure relative to MAPopt was not associated with thalamic injury.
Liu et al. (2021)	79 neonates (59 percent male) Johns Hopkins Neonatal Intensive Care Unit	Investigated whether the wavelet hemoglobin Volume index (wHVx) would identify MAPopt, and that blood pressures closer to MAPopt would be associated with less brain injury on MRI.	Hypoxic–ischemic encephalopathy	NIRS (INVOS 5100; neonatal probes), Medtronic	Wavelet hemoglobin volume index (wHVx) and hemoglobin volume index (HVx) were used to derive MAPopt. Derivation of MAPopt was continuous.	HVx or wHVx values were averaged and sorted into 3 mmHg bins of MAP to generate bar graphs. An automatic curve fitting method generated a U‐shaped curve with MAPopt at the nadir. When a nadir in HVx or wHVx was not found, MAPopt was declared unidentifiable. Both single window (3‐hour window) and multi‐window (twelve 2–4 h overlapping windows) weighted methods were used to derive MAPopt values.	MAP exceeding MAPopt was correlated with reduced injury in critical brain regions such as the paracentral gyri (*p* = 0.044), basal ganglia (*p* = 0.015), thalamus (*p* = 0.013), and brainstem (*p* = 0.041). MAP below MAPopt was not associated with regional brain injury.
Tekes et al. (2015)	31 neonates Johns Hopkins University	Investigated whether the lower Apparent diffusion coefficient (ADC) values would correlate with worse autoregulatory Function or not in neonates with perinatal hypoxic–ischemic injury	Neonatal hypoxic–ischemic injury	NIRS Neonatal cerebral oximetry probes (INVOS), Medtronic	Hemoglobin volume index (HVx) was used to derive MAPopt. Derivation of MAPopt was a single‐point measure.	Right and left HVx values were averaged and sorted into 5 mmHg bins of MAP to generate bar graphs. Bin with the most negative HVx was identified as MAPopt in each time period. Visual inspection was used to identify MAPopt values.	During hypothermia and rewarming period, neonates with 10 days or older who had MRI, decreased diffusion scores at Posterior centrum semiovale (*p* < 0.006) and the posterior limb of internal capsule (*p* = 0.04) were correlated with blood pressure deviation below MAPopt.

Abbreviations: CVR, cerebrovascular reactivity; NIRS, near infrared spectroscopy; MAPopt, mean arterial pressure optimum.

**TABLE 2:** Studies documenting statistical association between MAPopt and clinical outcome for the cardiac arrest population.

Article	Patient cohort characteristics and study site	Primary objective of study	Underlying pathology	Technology used for CVR assessment	CVR index used for MAPopt derivation	MAPopt derivation process	MAPopt documented association with clinical outcome
Griesdale et al. (2020)	59 patients (22% female) Vancouver General Hospital (Vancouver, BC, Canada), St. Paul's Hospital (Vancouver, BC, Canada), and Sunnybrook Health Sciences Center (Toronto, ON, Canada)	Assessing Cerebral Autoregulation and Optimal Mean Arterial Pressure in Patients with Hypoxic–Ischemic Brain Injury	Cardiac arrest	NIRS INVOS oximeter, Medtronic	Cerebral oximetry index (COx) was used to derive MAPopt. MAPopt was calculated using the “OptimalValueFlex” function in the ICM+ software. Derivation of the MAPopt was continuous.	MAPopt was calculated using a multi‐window weighted algorithm (ICM+ “OptimalValueFlex” function). This algorithm generated a U‐shaped curve plotted through the COx values across 5 mm Hg bins of MAP per patient. Based on the weighing approach, the summative MAPopt was generated. Instead of a single window, this method used data from 36 windows of between 2 and 8 h in duration.	No difference between favorable and unfavorable outcomes based on MAPopt.
Hori et al. (2016)	99 patients (mean age 65 years; 61.6 percent male) Johns Hopkins University	Evaluating whether excursions of blood pressure from the optimal mean arterial pressure during and after cardiac surgery are associated with postoperative delirium	Patients undergoing cardiac surgery	Ultrasound tagged NIRS UT‐NIRS monitoring was performed using a CerOx (cerebral oxidation) monitor	Correlation flow index (CFx) was used to derive MAPopt. Derivation of MAPopt was a single‐point measure.	CFx was categorized and averaged in 5 mmHg MAP bins for each patient. MAPopt was defined as the MAP in which CFx was lowest. The right‐ and left‐sided MAPopt values were averaged to define the individual MAPopt.	Blood pressure excursions below or above MAPopt were not associated with delirium in postoperative day 1 and 3, but blood pressure excursions above MAPopt were associated with delirium in postoperative day 2 (*p* = 0.011). Among the delirium subtypes, there were no significant differences in blood pressure excursions below or above MAPopt.
Kirschen et al. (2022)	22 patients (14 male) University of Pennsylvania	Investigated whether deviations from PRx‐derived optimal mean arterial pressure (MAPopt) were associated with in‐hospital mortality after adult cardiac arrest.	Cardiac arrest	Intracranial pressure (ICP) monitoring	Pressure reactivity index (PRx) was used to derive MAPopt. Derivation of MAPopt was continuous.	MAPopt was determined over time using a multi‐window approach. The authors plotted PRx versus MAP and used an automated curve fitting algorithm to fit a second‐order polynomial representing a convex parabola. The nadir of the fitted curve represented MAPopt. Parabolic curves from different windows were combined using a weighted average to determine the overall MAPopt every minute.	Higher deaths occurred for patients who had a higher burden of blood pressure, more than 5 mmHg below MAPopt. No difference in survival was found for patients with higher blood pressure burden 5 mmHg above MAPopt. But PRx‐derived MAPopt values did not differ between survivals and non‐survivals (*p* = 0.64).
Kirschen et al. (2021)	34 children (median age 2.9 years; 71 percent male) Children hospital of Philadelphia pediatric intensive care unit	Evaluating cerebrovascular autoregulation (CAR) using near‐infrared spectroscopy (NIRS) after pediatric cardiac arrest and determining if deviations from CAR‐derived optimal mean arterial pressure (MAPopt) are associated with outcomes	Cardiac arrest	NIRS Either unilateral or bilateral cerebral NIRS connected to integrative multimodality neuromonitoring device (Moberg Research)	Cerebral oximetry index (COx) was used to derive MAPopt. Derivation of MAPopt was continuous.	MAPopt was determined over time using a multi‐window approach. The authors plotted COx versus MAP in 5 mmHg bins and used an automated curve fitting algorithm to fit a second‐order polynomial representing a convex parabola. The nadir of the fitted curve represented MAPopt. The Fisher transform was applied to binned COx values to avoid ceiling effects. Parabolic curves from different windows were combined using a weighted average to determine the overall MAPopt every minute.	Patients with unfavorable outcomes spent more time with MAP below MAPopt −5 mmHg and had a greater difference between MAP and MAPopt during the first 24 h after cardiac arrest. Burden of MAP less than MAPopt −5 mmHg was greater for unfavorable outcomes versus favorable outcomes (*p* = 0.01).
Lee et al. (2014)	36 children (53 percent male) Johns Hopkins Pediatric Intensive Care Unit	Evaluating the relationship between cerebrovascular autoregulation and neurologic outcomes after pediatric cardiac arrest	Cardiac arrest	NIRS Pediatric cerebral oximetry sensors (INVOS), Medtronic	Hemoglobin volume index (HVx) was used to derive MAPopt. Derivation of MAPopt was a single‐point measure.	Right and left HVx values were averaged and sorted into 5 mmHg bins of MAP to generate bar graphs. Bin with the most negative HVx was identified as MAPopt in each time period. MAPopt was identified in several time periods (first 12 h, first 24 h, second 24 h, third 24 h, first 48 h, and first 72 h after ROC). Visual inspection was used to identify MAPopt values.	During the first 48 h, greater time spent in blood pressure below MAPopt was associated with primary neurologic death in children without extracorporeal membrane oxygenation (ECMO). After the return of circulation during the second 24 h, more time spent in blood pressure below MAPopt was associated with receiving a tracheostomy/gastrostomy in children without ECMO.

Abbreviations: CVR, cerebrovascular reactivity; ECMO, extracorporeal membrane oxygenation; MAPopt, mean arterial pressure optimum; NIRS, near‐infrared spectroscopy; ROC, return of circulation.

**TABLE 3:** Studies documenting statistical association between MAPopt and clinical outcome for the general ICU population.

Article	Patient cohort characteristics and study site	Primary objective of study	Underlying pathology	Technology used for CVR assessment	CVR index used for MAPopt derivation	MAPopt derivation process	MAPopt documented association with clinical outcome
Khan et al. (2024)	42 patients Queen's University	Characterizing optimal mean arterial pressure (MAPopt) ranges in critically ill adults without brain injury and determining whether deviations from these targets contribute to ICU delirium	Critically ill patients without brain injury	NIRS ForeSightTM near‐infrared spectroscopy oximeter (Edwards Lifesciences)	Cerebral oximetry index (COx) was used to derive MAPopt. MAPopt was calculated using custom algorithms developed in R and Python. Derivation of MAPopt was a single‐point measure.	MAPopt was calculated as mean MAP for which COx = 0, where ±1 standard deviation (SD) represents upper and lower MAPopt limits.	MAPopt values were not significantly different based on the history of hypertension or delirium. Additionally, delirium was not associated with deviations from MAPopt. There were no significant differences in MAPopt between “ever” delirious and “never” delirious patients (*p* = 0.18)
Zhang et al. (2024))	15 patients (33.3 percent female) At a tertiary academic hospital	Determining the feasibility of continuous CA monitoring in adult ECMO patients	Patients underwent ECMO cannulation in the intensive care unit.	NIRS (INVOS 5100 C; Medtronic)	Cerebral oximetry index (COx) was used to derive MAPopt. Derivation of MAPopt was continuous.	MAPopt values for each individual patient were determined at 6‐h intervals by placing MAP values in 5 mmHg bins and identifying the MAP associated with the lowest COx	Patients with good neurological outcome at 3 and 6 months spent less time outside of MAPopt than patients with poorer outcome at 3 and 6 months (74% vs. 82%, *p* = 0.01).

Abbreviations: CVR, cerebrovascular reactivity; MAPopt, mean arterial pressure optimum; NIRS, near‐infrared spectroscopy.

**TABLE 4:** Studies documenting statistical association between MAPopt and clinical outcome for the neurological ICU population.

Article	Patient cohort characteristics and study site	Primary objective of study	Underlying pathology	Technology used for CVR assessment	CVR index used for MAPopt derivation	MAPopt derivation process	MAPopt documented association with clinical outcome
Rivera‐Lara et al. (2019)	85 patients (mean age 57.7 years; 51.8 percent male) Johns Hopkins Hospital	Investigated whether comatose patients with greater duration and magnitude of clinically observed mean arterial pressure outside optimal mean arterial blood pressure have worse outcomes than those with mean arterial blood pressure closer to optimal mean arterial blood pressure	Comatose patients with acute neurologic disease	NIRS NIRS INVOS 5100, Medtronic	Cerebral oximetry index (COx) was used to derive MAPopt. Derivation of MAPopt was a single‐point measure.	MAPopt was calculated by averaging MAPopt from right and left sides. To calculate left and right MAPopt, MAP was plotted on the x‐axis in 5 mmHg bins and left or right COx was plotted on the y‐axis. MAPopt was defined as the MAP associated with the lowest COx. The authors calculated MAPopt in 24‐h windows and then calculated a weighted average by considering the number of hours monitored each day.	Patients with a difference greater than 10 mmHg between clinically observed mean arterial pressure and MAPopt (*p* = 0.013) and patients who spent more than 80 percent of the time outside of MAPopt (*p* = 0.04) were associated with mortality at 3 months. Similarly, both these criteria were associated with mortality at 6 months (*p* = 0.032 and *p* = 0.011, respectively).

Abbreviations: CVR, cerebrovascular reactivity; MAPopt, mean arterial pressure optimum; NIRS, near‐infrared spectroscopy.

**TABLE 5:** Studies documenting statistical association with cerebral multimodal monitoring.

Article	Patient cohort characteristics and study site	Primary objective(s) of the study	Pathological condition	Technology used for CVR assessment	CVR index to derive MAPopt/methods to derive MAPopt	MAPopt derivation process	Relationship between MAPopt and multimodal monitoring variables
Sekhon et al. (2019)	10 patients (7 male) Vancouver General Hospital ICU	(1) Characterize brain oxygenation and determine the prevalence of brain hypoxia, (2) Characterize autoregulation using the pressure reactivity index and identify the optimal mean arterial pressure, and (3) Assess the relationship between optimal mean arterial pressure and brain tissue oxygenation	Hypoxic ischemic brain injury after cardiac arrest	Intracranial pressure (ICP) monitoring	PRx (Pressure‐reactivity index) was used to derive MAPopt. Derivation of MAPopt was continuous (*OptimalValueFlex* function in ICM+).	MAPopt was determined as a secondary derivative by plotting PRx (*y*‐axis) against the MAP range (*x*‐axis) in 5 mm Hg bins. An automatic multi‐window curve‐fitting methodology was used to estimate MAPopt (*OptimalValueFlex* function in ICM+).	A nonlinear relationship was found between brain tissue oxygenation (PbtO_2_) and MAPdiff (MAP‐MAPopt). When the actual MAP was more extreme than –10 mm Hg below MAPopt, there was a linear rise of PbtO_2_ as MAPdiff approached –10 mm Hg. Between MAPdiff of −10 to +10 mm Hg, there was a less steep rise in PbtO_2_, with this relationship becoming more attenuated at the top of this range. MAPdiff above +10 mm Hg was not associated with further improvements in PbtO_2_. This relationship highlights that perfusion within the proximity of MAPopt is associated with improved PbtO_2_.

Abbreviations: CVR, cerebrovascular reactivity; MAPopt, mean arterial pressure optimum.

### 3.3 MAPopt versus clinical outcome

In general, across the included studies documenting formal objective comparison between MAPopt values and clinical outcomes, spending most of the time slightly above MAPopt was associated with good outcomes, while spending more time below MAPopt or well above MAPopt was associated with poor clinical outcomes. In the subsections to follow, subpopulations of neonatal hypoxic ischemic encephalopathy, cardiac arrest, general ICU, and neurological ICU are covered in more detail.

#### 3.3.1 Neonatal hypoxic ischemic encephalopathy population

All the listed neonatal population studies (Table 1) used NIRS for CVR assessment and hemoglobin volume index (HVx) to derive MAPopt. Of all the neonatal hypoxic ischemic encephalopathy studies, Burton et al. (2015) found that neonates who spent more time with MAP below MAPopt and those who had greater blood pressure deviation below MAPopt during the rewarming period were more likely to develop cognitive impairments at the age of 2 (*n* = 9; *p* = 0.048) (Burton et al., 2015). Chavez‐Valdez et al. (2017) found that greater maximal blood pressure deviation below MAPopt during normothermia was associated with a longer ICU stay for boys (*β* = 0.024, *p* = 0.018), but a shorter ICU stay for girls (*β* = −0.024, *p* = 0.024) (Chavez‐Valdez et al., 2017). These results indicate that gender may play a role in the clinical outcome. Howlett et al. (2013) found that spending time with MAP above MAPopt was associated with fewer injuries in different brain regions, particularly in the paracentral gyri, white matter, basal ganglia, and thalamus (Howlett et al., 2013). This study also found that patients with smaller changes in MAPopt between hypothermia and rewarming displayed better outcomes in the future. Liu et al. (2021) also found that spending time with MAP above MAPopt was associated with less injury in the paracentral gyri (*p* = 0.044), thalamus (*p* = 0.013), basal ganglia (*p* = 0.015), and brainstem (*p* = 0.041), but spending time with MAP below MAPopt did not have any negative impact on future outcome (Liu et al., 2021). However, in a different study, Lee, Perin, et al. (2017) found that not only was the time spent above or below MAPopt important, but also the grooming conditions of neonates (e.g., rewarming and hypothermia) determined physiological outcome (Lee, Perin, et al., 2017). Another study published in the same year by the same group found that greater duration and deviation of MAP below MAPopt were associated with negative outcomes in white matter (deviation below: *p* = 0.047) and paracentral gyri (greater duration: 0.007; deviation below: 0.012), but spending time with MAP within the MAPopt range resulted in less injury in white matter (*p* = 0.017) and basal ganglia (*p* = 0.04) (Lee, Poretti, et al., 2017). In an MRI study, Tekes et al. (2015) found that blood pressure deviation below MAPopt in neonates was correlated with decreased MRI diffusion scores in the posterior centrum semiovale (*p* < 0.006) and the posterior limb of the internal capsule (*p* = 0.04) (Tekes et al., 2015). Overall, neonatal studies found that spending time with MAP below MAPopt is associated with negative clinical outcomes, but gender and grooming conditions also played a crucial role in future clinical outcomes.

#### 3.3.2 Cardiac arrest population

All the listed cardiac arrest studies except one (Kirschen et al., 2022) (Table 2) used NIRS for CVR assessment. The other study (Kirschen et al., 2022) used intracranial pressure (ICP) monitoring for CVR assessment. Among the cardiac arrest studies, Griesdale et al. (2020) did not find any role of MAPopt on clinical outcomes (Griesdale et al., 2020). Cerebral oximetry index (COx) was used to derive MAPopt in this study. Kirschen et al. (2022) found that the mortality rate increased if patients spent more time below MAPopt −5 mmHg, but it did not matter for patients who had MAP above MAPopt +5 mmHg (Kirschen et al., 2022). Another study by this group Kirschen et al. (2021) found similar results (Kirschen et al., 2021). This study also found that the burden of MAP less than MAPopt‐5 mmHg was greater for unfavorable outcomes than favorable outcomes (*p* = 0.01) (Kirschen et al., 2021). Kirschen et al. (2021) used COx to derive MAPopt, whereas Kirschen et al. (2022) used pressure reactivity index (PRx) to derive MAPopt. In another study, Hori et al. (2016) found that blood pressure excursions below or above MAPopt were not associated with delirium on postoperative day 1 and 3, but blood pressure excursions above MAPopt were associated with delirium on postoperative day 2 (*p* = 0.011) (Hori et al., 2016). Among delirium subtypes, there was no significant difference in blood pressure excursions above or below MAPopt. In this study, Hori et al. (2016) used ultrasound‐tagged NIRS for CVR assessment, and, unlike any other studies discussed in this paper, this study used correlation flow index (CFx) to derive MAPopt. In another cardiac arrest study, Lee et al. (2014) found that during the first 48 h after cardiac arrest, spending more time below MAPopt was associated with neurologic death (Lee et al., 2014). Lee et al. (2014) used hemoglobin volume index (HVx) to derive MAPopt in this study. Even though the findings from cardiac arrest studies had mixed results, most of these studies found that spending more time with MAP below MAPopt is associated with worse clinical outcomes.

#### 3.3.3 General ICU population

Both general ICU studies (Table 3) used NIRS for CVR assessment and used COx to derive MAPopt. Among general ICU studies, Zhang et al. (2024) found that patients with good neurological outcomes at 3 and 6 months spent less time outside of MAPopt than patients with poorer outcomes at 3 and 6 months (74% vs. 82%, *p* = 0.01) (Zhang et al., 2024). In another general ICU study, Khan et al. (2024) did not find a straightforward relation between MAPopt and delirium (Khan et al., 2024). MAPopt values were not significantly different based on the history of hypertension or delirium. Delirium was not associated with deviations from MAPopt.

#### 3.3.4 Neurological ICU population

Our included only neurological ICU study (Table 4) used NIRS for CVR assessment. Rivera‐Lara et al. (2019) found similar results to those of Zhang et al. (2024). Patients who spent more than 80 percent of the time outside the range of MAPopt had a higher mortality rate at 3 and 6 months (*p* = 0.04 and *p* = 0.011, respectively) (Rivera‐Lara et al., 2019). This study used COx to derive MAPopt.

### 3.4 MAPopt versus MMM

Sekhon et al. (2019) was the only study we found that showed a relation between MAPopt and a multimodal monitoring variable through objective statistical analysis. This study (Table 5) used ICP monitoring for CVR assessment and pressure‐reactivity index (PRx) for MAPopt derivation. This study found a nonlinear relationship between brain tissue oxygenation (PbtO_2_) and deviation between MAP and MAPopt (Sekhon et al., 2019). PbtO_2_ linearly rose between more negative MAPopt and −10 mmHg MAPopt. Between −10 mmHg and +10 mmHg, there was a less steep rise of PbtO_2_ (Sekhon et al., 2019). Beyond that, PbtO_2_ did not change much as MAPopt increased (Sekhon et al., 2019). This relationship highlights that perfusion within the proximity of MAPopt is associated with improved PbtO_2_. This is the only study we found that shows the relation between MAPopt and a MMM variable.

Across the 15 included studies evaluating MAPopt and clinical outcomes, as well as the single study comparing MAPopt with MMM variables, the derivation methods for MAPopt varied substantially. Most studies in the neonatal population used near‐infrared spectroscopy (NIRS) combined with the hemoglobin volume index (HVx), relying on discrete time periods and visual inspection of binned data to determine the MAP with the most negative HVx. These were generally single‐point derivations. A few studies, particularly in the cardiac arrest populations, employed continuous derivation using automated, curve‐fitting algorithms (e.g., OptimalValueFlex in ICM+, polynomial fitting) applied to indices such as COx or PRx. Some studies used multi‐window, weighted average approaches to determine MAPopt dynamically across time (Griesdale et al., 2020; Kirschen et al., 2021, 2022; Sekhon et al., 2019). These differences in methodology—single‐point versus continuous, manual versus automated, and use of various cerebrovascular reactivity (CVR) indices—highlight methodological heterogeneity in MAPopt derivation.

## 4 DISCUSSION

Through this systematically conducted scoping review, we aimed to highlight the existing literature with objective documentation of: A. the association between MAPopt and clinical outcomes, and B. the association between MAPopt and MMM cerebral physiological variables/measures. The existing knowledge gaps related to MAPopt were highlighted. Currently, the existing MAPopt literature with objective outcome documentation remains highly heterogeneous, based on both methods of MAPopt derivation, patient populations studied, and the strength of outcome association. Similarly, there exists little literature to date that directly compares MAPopt to other MMM‐based cerebral physiology. In the sub‐sections below, we expand on some of the more nuanced discoveries based on subpopulation and study type.

### 4.1 Neonatal population

Studies involving neonates with hypoxic–ischemic encephalopathy consistently demonstrated that blood pressure deviations below MAPopt were associated with worse outcomes, including greater neurological impairment and increased brain injury in critical regions such as white matter, paracentral gyri, basal ganglia, and thalamus (Burton et al., 2015; Chavez‐Valdez et al., 2017; Howlett et al., 2013; Lee, Perin, et al., 2017; Lee, Poretti, et al., 2017; Liu et al., 2021; Tekes et al., 2015). This association appears anatomically specific, suggesting regional vulnerability to cerebral hypoperfusion. Additionally, the stability of MAPopt between hypothermia and rewarming phases emerged as a potential predictor of better outcomes (Howlett et al., 2013), indicating that the dynamic nature of cerebral autoregulation during therapeutic temperature management should be considered when determining hemodynamic targets.

Interestingly, Chavez‐Valdez et al. (2017) observed gender‐specific associations between blood pressure relative to MAPopt and clinical outcomes, suggesting potential sex‐dependent differences in cerebrovascular physiology that warrant further investigation (Chavez‐Valdez et al., 2017). These findings underscore the complexity of cerebral autoregulation in developing brains and highlight the need for individualized approaches to blood pressure management in neonatal critical care.

### 4.2 Cardiac arrest and cardiac surgery patients

In adult and pediatric patients after cardiac arrest, the relationship between MAPopt and outcomes was generally consistent with neonatal findings, though with some variability. Kirschen et al. (2021) and Kirschen et al. (2022) demonstrated that spending more time with blood pressure below MAPopt ‐5 mmHg was associated with unfavorable outcomes and higher mortality (Kirschen et al., 2021, 2022). However, blood pressure excursions above MAPopt did not consistently correlate with clinical outcomes, suggesting a potential asymmetric risk profile where hypoperfusion may be more detrimental than hyperperfusion.

Two of the reviewed studies (Griesdale et al., 2020; Kirschen et al., 2022) did not demonstrate an association between absolute values of MAPopt and survival, suggesting that the absolute value of MAPopt may be less important than maintaining blood pressure relative to the individualized autoregulatory target. This concept emphasizes the importance of continuous monitoring and dynamic adjustment of blood pressure targets rather than targeting a specific predetermined value.

### 4.3 General and neurological ICU patients

In the general ICU setting, Zhang et al. (2024) found that patients with good neurological outcomes spent significantly less time outside MAPopt ranges, reinforcing the importance of maintaining blood pressure within autoregulatory boundaries (Zhang et al., 2024). However, Khan et al. (2024) did not identify a clear relationship between MAPopt and delirium in critically ill patients without brain injury, highlighting potential limitations in the applicability of cerebral autoregulation monitoring for certain neurological complications (Khan et al., 2024).

The included study in neurological ICU patients provided robust evidence for the clinical relevance of MAPopt. Rivera‐Lara et al. (2019) demonstrated that both the magnitude and duration of blood pressure deviations from MAPopt were associated with mortality at 3 and 6 months in comatose patients with acute neurological disease (Rivera‐Lara et al., 2019). These findings suggest cerebral autoregulation‐guided blood pressure management may benefit patients with primary neurological conditions.

### 4.4 MAPopt and MMM variables

The limited number of studies examining the relationship between MAPopt and other physiological parameters represents a significant gap in our current understanding. This likely stems from the fact that most studies evaluating MAPopt are in non‐TBI populations, where invasive cerebral physiologic monitoring is not used, and thus most MMM raw and derived measures are unavailable. The single study by Sekhon et al. (2019) identified a nonlinear relationship between brain tissue oxygenation (PbtO_2_) and the difference between actual MAP and MAPopt (Sekhon et al., 2019). This complex relationship suggests that perfusion within proximity of MAPopt is associated with improved PbtO_2_. This finding has important implications for clinical practice, as it suggests that simply increasing blood pressure may not always improve cerebral oxygenation and could potentially be detrimental if pushed too far beyond autoregulatory capacity. However, the physiological significance of the findings of this study requires further evaluation.

### 4.5 Limitations of this review

This scoping review has several limitations that should be considered when interpreting its findings.

1. First, there is marked heterogeneity in MAPopt derivation methods across the included studies. Studies varied widely in the CVR indices used to derive MAPopt (e.g., HVx, COx, PRx, CFx, and wHVx), the technologies employed (mostly NIRS, but also invasive monitoring), and in the analytical approach—ranging from manual visual inspection of bar graphs to continuous automated curve fitting using multi‐window algorithms. Some studies calculated MAPopt as single‐point estimates, while others derived it continuously over time. These methodological inconsistencies limit comparability across studies and hinder the generalization of findings. Without standardized derivation approaches, it becomes challenging to draw reliable conclusions about the clinical utility of MAPopt or to assess its association with outcomes across different populations and contexts.
2. Second, all the studies included were observational, limiting causal inferences regarding the relationship between blood pressure management relative to MAPopt and clinical outcomes. The absence of randomized controlled trials explicitly targeting MAPopt prevents definitive conclusions about the efficacy of autoregulation‐guided therapy compared to standard care.
3. Third, the small sample sizes in many studies reduce statistical power and increase the risk of type II errors. This is particularly relevant for subgroup analyses and may explain inconsistent findings across studies.
4. Fourth, the extreme variance in nomenclature and taxonomy of various MAPopt metrics and related MMM cerebral physiological measures makes generating a comprehensive search strategy a challenge. This is highlighted by our identification of additional studies from the reference sections of the final included studies.
5. Fifth, by requiring objective statistical comparisons between MAPopt data and either clinical outcome or MMM physiology, many general studies on MAPopt derivation were not eligible to be included in this review. This highlights a limitation of current literature in the field.
6. Lastly, publication bias cannot be excluded, as studies with negative or null findings regarding the relationship between MAPopt and outcomes may be less likely to be published. The limited number of studies (only one study) exploring the association between MAPopt and multimodal monitoring variables highlights a significant gap in the literature that warrants further investigation. This likely stems from the patient populations in which most MAPopt studies are conducted (i.e., non‐TBI), where invasive cerebral physiologic monitoring (and thus most MMM raw and derived metrics) is not available or utilized.

### 4.6 Clinical implications and future directions

This review provides evidence that deviations of blood pressure from MAPopt values are associated with clinical outcomes in the patient population. It also showed that NIRS was the predominant technology used across studies, highlighting its feasibility as a noninvasive tool for continuous monitoring of cerebrovascular reactivity. Various indices derived from NIRS, including hemoglobin volume index (HVx) and cerebral oximetry index (COx), have demonstrated utility in identifying MAPopt and guiding blood pressure management. However, several challenges must be addressed before the clinical application of MAPopt can be considered. Essential steps include standardizing monitoring techniques, determining optimal indices for different patient populations, and developing user‐friendly bedside systems for real‐time analysis with reliable high‐yield continuous algorithms. Additionally, prospective randomized controlled trials are needed to establish whether targeting MAPopt improves clinical outcomes compared to standard blood pressure management. Future research should focus on several key areas, including elucidating the relationship between MAPopt and other physiological parameters, including cerebral blood flow, metabolism, and electrical activity, validating the feasibility of deriving MAPopt using NIRS cerebral autoregulatory metrics, and generating a custom algorithm for continuous derivation of MAPopt using NIRS‐based metrics.

## 5 CONCLUSION

This systematic scoping review provides evidence supporting the clinical relevance of MAPopt/ABPopt in various patient populations. There are consistent associations between blood pressure deviations from MAPopt and clinical outcomes. Specifically, maintaining blood pressure above or within individualized MAPopt ranges appears to be associated with improved outcomes, while blood pressure below MAPopt correlates with poorer outcomes. There exists a lack of objective literature examining the relationship between MAPopt/ABPopt and other MMM cerebral physiology. Future research is required to improve MAPopt/ABPopt algorithm calculation efficiency, so that directed studies on MMM physiological relationships and clinical outcomes can be prospectively conducted.

## FUNDING INFORMATION

This work was directly supported through the Endowed Manitoba Public Insurance (MPI) Chair in Neuroscience and the Natural Sciences and Engineering Research Council of Canada Alliance Advantage program (NSERC; ALLRP‐597708‐24; Medtronic External Research Program Grant ERP‐2024‐14025).

## CONFLICT OF INTEREST STATEMENT

F.A.Z. currently has NSERC Alliance Advantage grant (ALLRP‐597708‐24) support in partnership with Medtronic's Acute Care & Monitoring Division (ERP‐2024‐14025) for work that is related to this manuscript. Funding from the partner organization is provided to match NSERC governmental funding only, in keeping with NSERC policies. Medtronic does not direct the research objectives, data collection, analysis, interpretation, or publication of the findings in any way. All other authors assert that they have no conflicts of interest regarding this work, confirming the absence of any financial interests, affiliations, or personal relationships that may have influenced or biased this research.

## GENERATIVE AI STATEMENT

The author(s) declare that no Generative AI was used in the creation of this manuscript.

## Supporting information


Data S1.

## Appendix

The detailed strings of keywords that were used as search parameters in BIOSIS, SCOPUS, EMBASE, MEDLINE, Global Health, and Cochrane Library were as follows:

### MAPopt versus neurological outcome

“MAPopt” OR “Mean Arterial Pressure optimization” OR “MAP opt” OR

“Mean arterial pressure optimal” OR “Mean arterial pressure optimum”

OR “Optimal mean arterial pressure” OR “Optimum mean arterial pressure”

“Blood pressure optimal” OR

“Blood pressure optimum” OR “MAP optimization” OR

“ABP opt” OR “ABPopt” OR “Arterial blood pressure optimum” OR

“Optimum arterial blood pressure” OR “arterial blood pressure optimal”

OR “Optimal arterial blood pressure” OR

“Arterial blood pressure optimization”

AND

“outcome” OR “Cognitive function” OR “Motor function” OR

“recovery” OR “Survival” OR “Disability” OR

“Death” OR “Prognosis” OR “Quality of life” OR

“Neurological function” OR “morbidity” OR

“mortality”

### MAPopt versus MMM variable

“MAPopt” OR “Mean Arterial Pressure optimization” OR “MAP opt” OR

“Mean arterial pressure optimal” OR “Mean arterial pressure optimum”

OR “Optimal mean arterial pressure” OR “Optimum mean arterial pressure”

“Blood pressure optimal” OR

“Blood pressure optimum” OR “MAP optimization” OR

“ABP opt” OR “ABPopt” OR “Arterial blood pressure optimum” OR

“Optimum arterial blood pressure” OR “arterial blood pressure optimal”

OR “Optimal arterial blood pressure” OR

“Arterial blood pressure optimization”

AND

“Multimodal monitoring” OR “Multifaceted monitoring” OR

“Cerebral oxygenation” OR “Brain oxygenation” OR

“Cerebral blow flow” OR “Brain surgery” OR “Critical care” OR

“Intracranial pressure” OR “Intercranial pressure” OR “Cerebral autoregulation”

OR “Neurophysiological” OR “Neurocritical care bioinformatics” OR

“Brain tissue oxygenation” OR “Brain tissue oxygen tension” OR

“Parenchymal brain tissue oxygen” OR “PbtO2” OR

“Jugular bulb venous oxygen saturation” OR “Jugular bulb monitoring”

OR “SjvO2” OR “Brain oxygen” OR “Cerebral oxygenation” OR

“Cerebral oxygen utilization” OR “Licox” OR

“Neurovent‐PTO” OR “Arterial‐jugular venous oxygen content difference” OR

“AVDO2” OR “Peripheral oxygen saturation” OR “SpO2” OR

“Near Infrared spectroscopy” OR “NIRS” OR

“Regional oxygen saturation” OR “Regional cerebral oximetry” OR

“rSO2” OR “Electroencephalogram” OR “EEG” OR

“Electroencephalography” OR “Electrocortical activity” OR

“Electrophysiology” OR “Electrophysiological” OR

“Cerebral microdialysis” OR “Cerebral metabolism” OR “Brain metabolism” OR

“Cerebral metabolites” OR “Brain metabolites” OR

“autoregulation” OR “Cerebrovascular reactivity” OR “Autoregulatory reserve” OR

“Vascular reactivity” OR “Cerebral vasoreactivity” OR “Pressure reactivity index” OR

“PRx” OR “Pulse amplitude index” OR “PAx” OR “RAC” OR

“Cerebral blood flow index” OR “CBFX” OR “CBFX‐a” OR

“Cerebral Oximetry index” OR “Cox” OR “Cox‐a” OR

“Diastolic flow index” OR “Dx” OR “Dx‐a” OR “Deoxyhemoglobin index” OR

“Hbx” OR “Hbx‐a” OR “Oxyhemoglobin index” OR “HbOx” OR

“HbOx‐a” OR “Hemoglobin volume index” OR “HVx” OR

“Induced pressure reactivity index” OR “iPRx” OR “Long Pressure reactivity index” OR

“L‐PRx” OR “Low‐frequency autoregulation index” OR “Lax” OR

“Laser‐Doppler index” OR “LDx” OR “Lx” OR “Lx‐a” OR

“Mean flow index” OR “Mx” OR “Mx‐a” OR “Oxygen reactivity index” OR

“ORx” OR “PRx55‐15” OR “Systolic flow index” OR “Sx” OR “Sx‐a” OR

“Total hemoglobin index” OR “THx” OR “THx‐a” OR “Tissue oxygen index” OR

“Tox” OR “Tox‐a” OR “Wavelet Cerebral oximetry index” OR “wCOx” OR

“Wavelet hemoglobin volume index” OR “wHVx” OR

“Wavelet Pressure Reactivity Index” OR “wPRx” OR

“Compensatory reserve” OR “RAP” OR “Mean arterial pressure” OR

“Medium arterial pressure” OR “systolic” OR “Blood pressure” OR

“MAP” OR “Cerebral blood flow” OR “Transcranial doppler” OR “TCD” OR

“Lindegaard ratio” OR “Critical closing pressure” OR

“Cerebrovascular time filling constant” OR “Pulsatility index” OR

“Transcranial color‐coded duplex sonography” OR “TCCS” OR

“Thermal diffusion” OR “Hemedex” OR “Bowman perfusion” OR

“Laser Doppler Flowmetry” OR “End‐tidal CO2” OR “EtCO2” OR

“Respiratory dynamics” OR “autonomics” OR “Heart rate variability” OR

“Bispectral” OR “BIS” OR “Heart rate” OR “Signal entropy” OR

“Multi‐scale entropy”
