# Supplementary material for: Optimal mean arterial pressure and its objective statistical associations with clinical outcomes and multimodal monitoring cerebral physiology: A systematic scoping review
Source: Physiol Rep. 2025 Sep 21;13(18):e70545. doi: 10.14814/phy2.70545 (PMC12451020; doi:10.14814/phy2.70545)
Supplement: Supplementary file 1 — Data S1. [file PHY2-13-e70545-s001.pdf]

Table: PRISMA ScR Checklist

Section	Item	PRISMA-ScR Checklist Item
**Title**	1	See pg. 1
**Abstract**		
Structured summary	2	See pg. 5 (Abstract)
**Introduction**		
Rationale	3	See pg. 5-6 (Section 1)
Objectives	4	See pg. 5-6 (Section 1)
**Methods**		
Protocol and registration	5	N/A
Eligibility criteria	6	See pg. 7-8 (Section 2.2)
Information sources	7	See pg. 8 (Section 2.3)
Search	8	See pg. 8 (Section 2.3)
Selection of sources of evidence	9	See pg. 8 (Section 2.4)
Data charting process	10	All relevant data were collected from each article by the primary author
Data items	11	See pg. 8 (Section 2.5)
Critical appraisal of individual sources of evidence	12	N/A
Summary measures	13	N/A
Synthesis of results	14	All data items for each method were tabulated and are included in Tables 1-5
Risk of bias across studies	15	As such, all articles published in academic journals were assumed to have been screened.
Additional analyses	16	N/A
**Results**		

Selection of sources of evidence	17	See pg. 9-33 (Section 3)
Characteristics of sources of evidence	18	See Tables 1-5
Critical appraisal of sources of evidence	19	N/A
Results of individual sources of evidence	20	See Tables 1-5
Synthesis of results	21	All articles included a valid relationship between MAPopt and outcome/MMM variables.
Risk of bias across studies	22	As such, all articles published in academic journals were assumed to have been screened.
Additional analysis	23	N/A
**Discussion**		
Summary of evidence	24	See pg. 33-35 (Section 4)
Limitations	25	See pg. 35-36 (Section 4.5)
Conclusions	26	See pg. 36 (Section 5)
**Funding**	27	See pg. 3-4 (Funding)
